# GLUT3-mediated cigarette smoke-induced epithelial-mesenchymal transition in chronic obstructive pulmonary disease through the NF-kB/ZEB1 pathway

**DOI:** 10.1186/s12931-024-02785-3

**Published:** 2024-04-09

**Authors:** Yu Ding, Ziteng Wang, Zheming Zhang, Rong You, Yan Wu, Tao Bian

**Affiliations:** Department of Respiratory Medicine, Wuxi People’s Hospital, Wuxi Medical Center, The Affiliated Wuxi People’s Hospital of Nanjing Medical University, Nanjing Medical University, Wuxi, Jiangsu 214023 People’s Republic of China

**Keywords:** GLUT3, NF-κB, ZEB1, Airway remodelling, COPD

## Abstract

**Background:**

Airway remodelling plays an important role in the pathogenesis of chronic obstructive pulmonary disease (COPD). Epithelial–mesenchymal transition (EMT) is a significant process during the occurrence of airway remodelling. Increasing evidence suggests that glucose transporter 3 (GLUT3) is involved in the epithelial mesenchymal transition (EMT) process of various diseases. However, the role of GLUT3 in EMT in the airway epithelial cells of COPD patients remains unclear.

**Methods:**

We detected the levels of GLUT3 in the peripheral lung tissue of COPD patients and cigarette smoke (CS)-exposed mice. Two Gene Expression Omnibus GEO datasets were utilised to analyse GLUT3 gene expression profiles in COPD. Western blot and immunofluorescence were used to detect GLUT3 expression. In addition, we used the AAV9-GLUT3 inhibitor to reduce GLUT3 expression in the mice model. Masson’s staining and lung function measurement were used detect the collagen deposition and penh in the mice. A cell study was performed to confirm the regulatory effect of GLUT3. Inhibition of GLUT3 expression with siRNA, Western blot, and immunofluorescence were used to detect the expression of E-cadherin, N-cadherin, vimentin, p65, and ZEB1.

**Results:**

Based on the GEO data set analysis, GLUT3 expression in COPD patients was higher than in non-smokers. Moreover, GLUT3 was highly expressed in COPD patients, CS exposed mice, and BEAS-2B cells treated with CS extract (CSE). Further research revealed that down-regulation of GLUT3 significantly alleviated airway remodelling in vivo and in vitro. Lung function measurement showed that GLUT3 reduction reduced airway resistance in experimental COPD mice. Mechanistically, our study showed that reduction of GLUT3 inhibited CSE-induced EMT by down-regulating the NF-κB/ZEB1 pathway.

**Conclusion:**

We demonstrate that CS enhances the expression of GLUT3 in COPD and further confirm that GLUT3 may regulate airway remodelling in COPD through the NF-κB/ZEB1 pathway; these findings have potential value in the diagnosis and treatment of COPD. The down-regulation of GLUT3 significantly alleviated airway remodelling and reduced airway resistance in vivo. Our observations uncover a key role of GLUT3 in modulating airway remodelling and shed light on the development of GLUT3-targeted therapeutics for COPD.

## Introduction

Chronic obstructive pulmonary disease (COPD) is a chronic airway inflammatory disease and the third leading cause of death in the world, causing over 3.23 million deaths. COPD has received a great deal of attention because of its high morbidity rate, the huge damage it causes, and the heavy burden on society [1–4]. Airway remodelling, which contributes to the important pathological changes in COPD, can lead to airway obstruction, irreversible airflow limitation, and a decline in pulmonary function [5]. Recent advances in remodelling the airways in COPD have attracted great attention due to its increasing prevalence.

Recently, several studies have found that EMT, epithelial-mesenchymal transformation, is a key process in airway remodelling [6]. EMT participated in a series of regulatory changes, causing epithelial cells to undergo a transformation and exhibit motility and mesenchymal-epithelial cell phenotype. An important manifestation of EMT involves the upregulation of specific key transcription factors (TFs), such as ZEB1, Snail, and Twist [7–9]. The process of EMT is accompanied by the dissolution of adhesive junction proteins. The disruption of connections, such as a decrease in the expression of E-cadherin, leads to the dissociation of epithelial cells. Meanwhile, the EMT process induced the expression of mesenchymal marker proteins, such as enhanced expression of N-cadherin and vimentin. Significantly, E-cadherin is a cell adhesion molecule and its main role in cells is to form and maintain cell-cell interactions. Therefore, the loss of E-cadherin expression is an important marker of EMT. Zinc finger E-box binding Homeobox 1 (ZEB1) is considered a transcription factor in the process of EMT. During the EMT process, it leads to the occurrence of EMT by binding to the E-box sequence of the CDH1 promoter region and inhibiting the expression of E-cadherin [9, 10]. EMT has been implicated in the airways in cell hypertrophy, metaplasia, modification of epithelium, and gene mutation [2]. Some studies have discovered that the EMT process of epithelial cells is closely associated with COPD’s pathogenesis [6, 7], and its potential mechanism needs further research.

Glucose transporter 3 (GLUT3), also known as SLC2A3, is an important member of the glucose transporter family [11]. Glucose transporters play a key role in taking up glucose from extracellular to intracellular, following the concentration gradient [12]. GLUT3 expression has been reported in gastric cancer, testicular cancer, ovarian cancer, and non-small cell lung cancer. Recently, a study showed that in a mouse adenocarcinoma model, inhibiting GLUT3 can inhibit tumour development [13]. The high expression of GLUT3 can serve as a poor prognostic indicator for patients with non-small cell lung cancer (NSCLC), promoting the occurrence and development of EMT [14–17]. Previous research has confirmed that GLUT3 can play an important role in the emergence of EMT [18]. In addition, studies have shown that glucose transporters have some effect on the epithelium of the airway [19]. Epithelial cells require energy to drive cell proliferation and are divided into secretions and surfactants, with glucose uptake an important energy source [20]. Our previous research has found that SLC2A3 had high expression in the lung tissue of patients with COPD. However, the role and specific molecular mechanisms of GLUT3 in airway remodelling in COPD are still unclear.

In this work, we analysed the GEO dataset and found that the expression of GLUT3 was higher in COPD patients than in the control group through bronchial mucosal biopsy. Thereafter, we detected an increase in GLUT3 expression both in vivo and in vitro under the stimulation of cigarette smoke and cigarette smoke-induced EMT. Mechanistically, GLUT3 regulated the occurrence of EMT by the NF-κB/ZEB1 pathway as a starting point in airway epithelial cells, exploring the molecular mechanisms of airway remodelling, COPD occurrence and development, and providing new ideas and therapeutic targets for identifying biomarkers of smoking-induced airway remodelling, and COPD prevention and treatment.

## Materials and methods

### Data acquisition

The published microarray datasets, GSE20257 (including 23 COPD and 53 control samples) and GSE10006 (including 27 COPD and 22 control samples) were received from the GEO database (https://www.ncbi.nlm.nih.gov/geo/) in the data format MINiML. We generated the above two datasets using GPL570 (HG-U133-Plus_2) Affimetrix Human Genome U133 Plus 2.0 array, both of which are gene expression arrays. Airway epithelial samples were isolated from the subjects with COPD samples and control groups in the GSE20257 and GSE10006 datasets. We pre-processed all GEO raw datasets using “affy” in R, including normalisation, background calibration and log2 conversion. When a common gene was mapped by multiple probes, the average value was taken as its expression value.

### Human samples

The peripheral lung tissue of non-smokers, smokers, and patients with COPD in this study was obtained from the lobectomy of benign pulmonary nodules and lung transplantation at Wuxi People’s Hospital. All patients with COPD met the diagnostic criteria of GOLD 2023. According to the Istanbul Declaration, all organisational donors in this study were voluntary, and informed consent was obtained from them. The Ethics Committee of Wuxi People’s Hospital approved the study protocol. After collecting peripheral lung tissue, it was deposited in a test tube and quickly stored at -80 ° C for subsequent use. The clinical information on the lung function of human lung tissue donors participating in this study is shown in Table 1.

**Table 1:** Clinical characteristics of the subjects

	Gender	Age	Smoking history (pack-years)	FEV_1_ (%) predicted	FEV_1_/FVC (%)	GOLD stages
Non smoker-1	male	46	–	106.9	87.39	–
Non smoker-2	male	51	–	72.8	86.48	–
Non smoker-3	male	79	–	90.4	83.17	–
Non smoker-4	male	60	–	80.5	92.44	–
Non smoker-5	male	58	–	82.5	80.07	–
Non smoker-6	male	63	–	81.6	86.75	–
Non smoker-7	male	65	–	81.4	98.44	–
Non smoker-8	male	72	–	74.9	72.76	–
Smoker-1	male	65	30	80.29	73.01	–
Smoker-2	male	53	30	118.1	84.81	–
Smoker-3	male	51	30	106.9	83.61	–
Smoker-4	male	69	40	119.8	86.77	–
Smoker-5	male	70	50	59.5	72.41	–
Smoker-6	male	70	45	87.2	92.62	–
Smoker-7	male	55	20	101.6	73.36	–
Smoker-8	male	76	20	86.6	83.27	–
COPD Smoker-1	male	77	40	46.2	52.73	
COPD Smoker-2	male	64	20	80.2	68.63	
COPD Smoker-3	male	63	40	85.3	62.01	
COPD Smoker-4	male	63	40	44.1	51.99	
COPD Smoker-5	male	79	40	26.6	29.49	
COPD Smoker-6	male	66	30	32.3	38.04	
COPD Smoker-7	male	66	35	46.5	36.44	
COPD Smoker-8	male	77	60	28.6	48.62	
	**Non Smoker**		**Smoker**		**COPD**	
Number	8		8		8	
Sex, male	8(100%)		8(100%)		8(100%)	
Age, years	61.75±10.66		63.6±9.3		69.4±7.0	
Smoking history, pack-years	–		33.1±11.0		38.1±11.3	
FEV_1_ (%) predicted	83.88±10.69		95.0±20.5		48.7±22.4	
FEV_1_/FVC (%)	85.9±7.7		81.2±7.5		48.5±13.3	

Data are presented as means ± SD, unless otherwise stated

### Animals and establishment of mice COPD model

C57BL6J mice (male, 6–8 weeks old) were raised in animal equipment at Wuxi People’s Hospital in Jiangsu Province and bought from Changzhou Kawensi Experimental Animal Co., Ltd. (China). The mice were treated humanely following the approval and guidelines of the Animal Care and Use Committee of Wuxi People’s Hospital in Jiangsu Province and the relevant laws on experimental animal management in Jiangsu Province. Mice were exposed to smoke from Daqianmen cigarettes (10 mg tar and 0.8 mg nicotine/cigarette, Shanghai, China). Mice were exposed to CS for 2 h, twice a day, with an interval of 4 h, 7 days a week, for 12 weeks in a tempered glass box, a systemic exposure system. In addition, these mice serve as controls in a safe and similar environment without exposure to CS-fed age-matched mice.

### Animals and administration of adenovirus

The method for establishing a mice COPD model has been described above. Genechem Technology Co., Ltd. (Genechem, China) synthesised the AAV9-GLUT3-inhibitor. The mice were divided into four groups (control, CS, CS + AAV negative control, and CS + AAV9-GLUT3-inhibitor). The virus titre was 5 × 10^11^ v.g./ml. Before mice were exposed to CS, we perfused them with adenovirus through the nose. The Animal Care approved all animal experiments, and the Use Committee of Wuxi People’s Hospital in Jiangsu Province according to the laws on experimental animal management in Jiangsu Province.

### Lung function measurement

At the Jiangsu Provincial Center for Disease Control and Prevention, whole-body plethysmography was used to measure lung function in mice (Buxko Electronics Co., Ltd., USA). We placed the mouse in a room connected to a sensitive baroreceptor and recorded the enhanced pause (Penh) using FinePoint software (Buxco Electronics, USA) when the mouse breathed peacefully. Penh mainly reflects the airway resistance of mice in the respiratory parameters recorded in the instrument. Take the average value and express it as an absolute Penh value.

### Regents and cell culture

BEAS-2B cells were derived from normal human pulmonary bronchial epithelial cells and received from the Chinese Academy of Science cell bank. BEAS-2B cells were cultured in Dulbecco’s modified Eagle’s medium (DMEM) supplemented with 10% foetal bovine serum (Gibco, USA), 100 U/mL penicillin, and 100 µg/mL streptomycin (Thermo Fisher Scientific, USA) in a humidified incubator containing 95% air and 5% CO2 at 37 °C. After the cells were 70–80% fused, they were digested with 0.25% trypsin (Gibco, USA) at 37 °C for 1–2 min. Observation under the microscope showed that most of the cells were in a single suspended state, and digestion was immediately terminated in the complete culture medium. The medium was then placed in the centrifuge at 1000 rpm for 5 min, the supernatant was discarded, and the cell precipitate was resuspended on the culture medium with the subculture at a 1:3 ratio. The cell generations used were 10–20 generations. Before modelling, the cells were seeded in a 6-well plate at 10^4^ cells per well. During exposure to CSE, the cells were cultivated in DMEM medium containing 10% FBS. Finally, we collected cells after exposure to CSE at 0 h, 6 h, 12 h, 24 h, and 48 h.

### Preparation of CSE

The method of preparing CSE was reported previously [8], and some modifications were made. Two cigarettes (Chinese front door) were dissolved in serum-free DMEM (Gibco, USA) with a pH of 7.4 to form a CSE solution. Then, a 0.22 µ M pore filter (Merck Microporous Company, USA) filtered and removed insolubility, standardised by measuring absorbance at 320 and 540 nm and defined as 100% CSE. We diluted CSE to 5% concentration using DMEM medium and used it immediately.

### Western blot

The total protein was extracted using RIPA buffer (Beyotime Institute of Biotechnology, China) mixed with a phosphatase inhibitor and protease inhibitor (CWBIO, China). Equal amounts of protein (20–40 µg) were separated via SDS‒PAGE and transferred onto PVDF membranes (Millipore, MA). After using 5% milk blocking for 1 h, the membranes were placed with antibodies targeting GAPDH monoclonal antibody 1:60000 (#60004-1-lg, Proteintech, China), GLUT3 polyclonal antibody 1:5000 (#20403-1-AP, Proteintech, China), E-Cadherin Mouse mAb 1:1000 (#14,472, Cell Signaling Technology, USA), ZEB1 polyclonal antibody 1:1000 (#21544-1-AP, Proteintech, China), N-cadherin XP Rabbit mAb 1:1000 (#13,116,Cell Signaling Technology, USA), Vimentin XP Rabbit mAb 1:1000 (#5741, Cell Signaling Technology, USA), and NF-κB XP Rabbit mAb 1:1000 (#8242, Cell Signaling Technology, USA) at 4 °C overnight. The membrane was then incubated with the secondary antibodies 1:3000 (Beyotime). The visualisation of antibodies was completed through the ECL system.

### Immunofluorescence staining

Fix cells in a 4% paraformaldehyde solution for 15 min, permeate 0.2% Tritunx-100 for 15 min, and wash with PBS three times. Then, the cells were sealed with cell sealing solution for 1.5 h and incubated overnight with primary antibodies (GLUT3 1:200, E-cadherin 1:200, N-cadherin 1:400, Vimentin 1:200) at 4 °C. Cells were incubated with fluorescent secondary antibodies (Abcam) and incubated at room temperature for 1 h, followed by DAPI for 5 min. Images were obtained using a confocal microscope.

### Immunohistochemistry staining

A paraffin slicer fixed the lung tissues with 4% paraformaldehyde and paraffin-embedded and sliced into 4-µm-thick lung sections. Briefly, lung sections were deparaffinised through graded alcohols and washed in distilled H_2_O. Heat-activated antigen retrieval was performed using a citrate buffer, and a peroxidase blocker blocked endogenous peroxidase. After that, sections were blocked for nonspecific protein binding by 10% normal serum. Sections were immunostained with anti-GLUT3 at a 1:500 dilution. After incubation with appropriate HRP-coupled secondary antibodies, the samples were incubated with DAB (Sigma, USA). Lung sections are observed under a microscope. A blinded reader quantified immunohistochemistry staining, and staining was quantified using the Threshold feature in ImageJ.

### Masson’s staining and H&E staining

The mouse right lung lobe and human lung tissue were fixed with 4% neutral paraformaldehyde for 24 h. Tissues were embedded in paraffin and sectioned (4 μm). The sample slides were stained with trichrome stain (Masson’s) kits (D026-1-2, Nanjing Jiancheng Bioengineering Institute, China) to detect collagen deposition according to the instructions. Next, a photograph documentation facility examined the slides under a light microscope. Using ImageJ software, collagen content was measured by the ratio of collagen’s surface area (blue) to the total surface area (red).

H&E staining was conducted according to the Hematoxylin-Eosin Staining Kit (Solarbio, G1120) manufacturer’s instructions.

### Small interfering RNA, plasmids, and transfection

An appropriate concentration of BEAS-2B Cells were planted in a 6-well culture plate and incubated overnight in a culture incubator. Three centrifuge tubes were taken and the appropriate Opti-MEM medium was added separately. For every 125 µl of Opti MEM medium in one tube, 3.75 µl Lipofectamine 3000 was added, after which we gently blew and sucked 3–5 times to mix well. An appropriate amount of siRNA or NC siRNA was added to each of the other two tubes, gently blowing and aspirating 3–5 times, to mix it well. Three centrifuge tubes were left to stand at room temperature for 5 min. The Opti-MEM medium was sucked out with the same volume of siRNA or NC siRNA tubes from centrifuge tubes containing Lipofectamine 3000. They were mixed separately and gently blown and sucked 3–5 times to mix well. After standing at room temperature for 20 min, an appropriate amount of the final mixture was taken and added to a 6-well plate to maintain the final concentration of siRNA or NC siRNA at 100 nM. Beas-2B cells transfected with siRNA were synthesised by RiBoBio (Guangzhou), targeting GLUT3, using liposome 3000 (Invitrogen, L3000-015) for 48 h. Human ZEB-1 expression vector was purchased from RiBoBio (Guangzhou). An appropriate concentration of beas-2b cells were taken and planted in a 6-well culture plate. The transfection procedures followed the manufacturer’s protocols. The original culture medium was discarded after 12 h, and complete culture medium was added to continue cultivation.

### Statistical analysis

All statistical analyses were conducted using GraphPad Prism 9 software (GraphPad Software Company, San Diego, CA) and displayed as the average ± SD of at least three independent experiments. Student’s t-test was applied for comparisons between the patient groups and was performed between two groups. The mean values were compared using the post hoc test Dunnet’s was used after one-way ANOVA when more than two groups were compared. A *P* < 0.05 value was considered statistically significant.

## Results

### GLUT3 expression was increased in airway epithelium, and airway remodelling occurred in patients with COPD

**Fig. 1:**
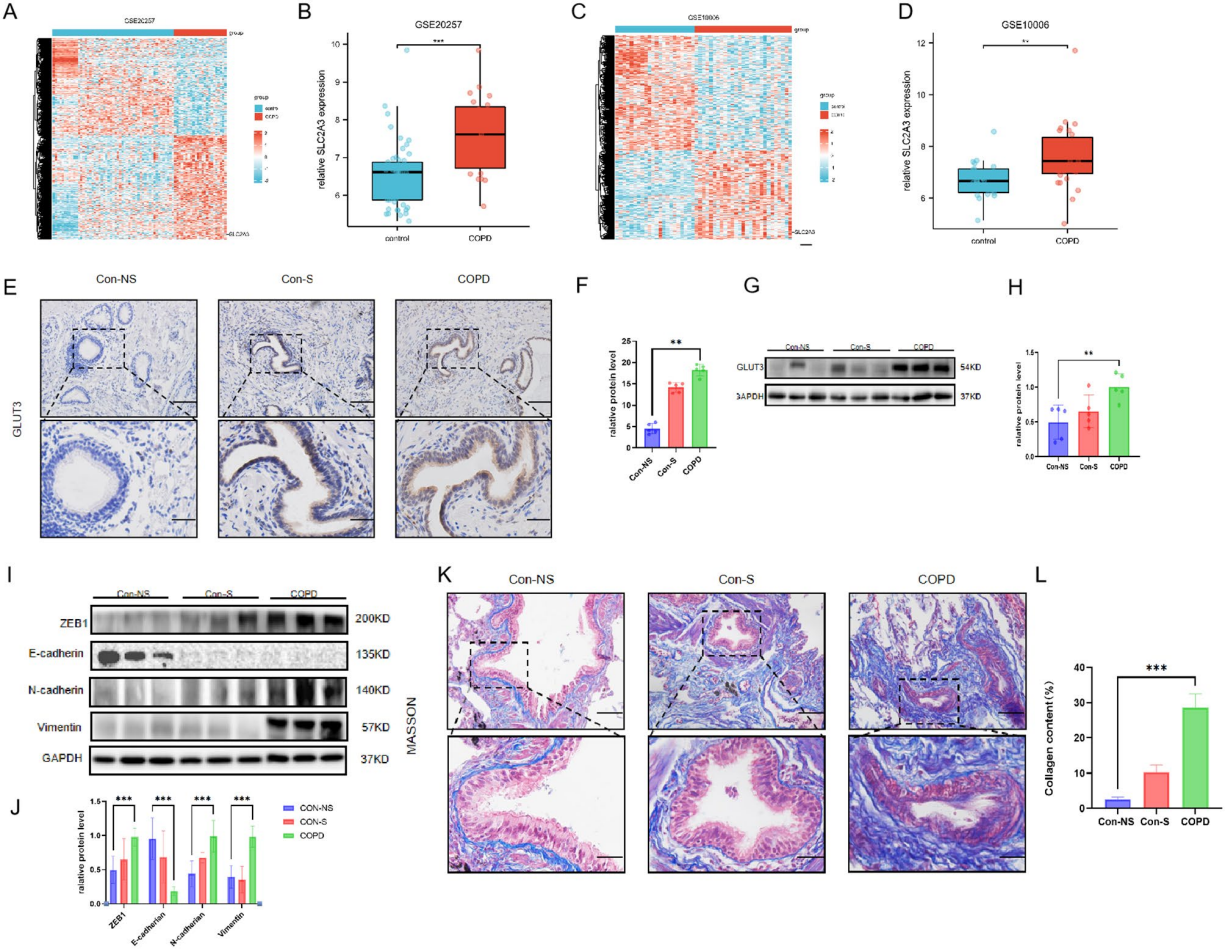
GLUT3 expression was increased in airway epithelium, and airway remodelling occurred in patients with COPD (**A**) and (**C**) The heat maps of GSE20257 and GSE10006. (**B**) and (**D**) The box plots of GSE20257 and GSE10006. (**E**) Representative airway GLUT3 immunohistochemical staining on lung sections of Con-NS, nonsmokers without COPD (*n* = 5), Con-S, smokers without COPD (*n* = 5), and COPD, COPD patients (*n* = 5). Scale bar, 100 μm (up). (**F**) Quantitative analysis of GLUT3 protein levels in airway epithelial cells. (**G**) and (**H**) The relative protein level of GLUT3 in the lung sections of Con-NS (*n* = 5), Con-S (*n* = 5), and COPD (*n* = 5) were determined by Western blot. (**I**) Western blots (**J**) The relative protein level of ZEB1, E-cadherin, N-cadherin, and Vimentin in the lungs of Con-NS (*n* = 8), Con-S (*n* = 8), and COPD (*n* = 8). (**K**) Perform Masson staining on lung sections to evaluate lung morphology. Scale bar, 100 μm. Collagen: blue, nucleus: black, cytoplasm/epithelial cells: red. (**L**) Quantification of Masson staining for collagen content from Con-NS (*n* = 3), Con-S (*n* = 3), and COPD patients (*n* = 3). The data are the mean ± SD. * *P* < 0.05, ** *P* < 0.01,*** *P* < 0.001

First, we searched the expression level of GLUT3 in COPD in the two GEO datasets. In GSE20257 and GSE10006, the expression of GLUT3 in the airway epithelial cells of COPD patients was higher than that in the control group, as shown in Fig. 1A, B, C, and D. The clinical characteristics and lung function of the subjects are shown in Table 1. We observed more intense GLUT3 staining in the peripheral lung tissue of patients with COPD compared with smokers and non-smokers (Fig. 1E and F). The expression of the GLUT3 protein (Fig. 1G) in the lung tissue also increased significantly in patients with COPD compared to smokers and non-smokers (Fig. 1H).

The airway remodelling caused by EMT in airway epithelial cells is a main process in smoking-induced COPD [6]. To indicate that cigarette smoke induces EMT, the expression of E-cadherin, which is the marker protein of epithelial cells, was down-regulated in the peripheral lung tissue of Patients with COPD. The expression of N-cadherin, Vimentin, and the core transcription factor ZEB1 were up-regulated(Fig. 1I and J). To determine whether airway remodelling is associated with COPD, Masson staining showed that patients with COPD had more collagen deposition than non-smokers and smokers (Fig. 1K and L).

### Airway remodelling was triggered in CS-induced experimental COPD

**Fig. 2:**
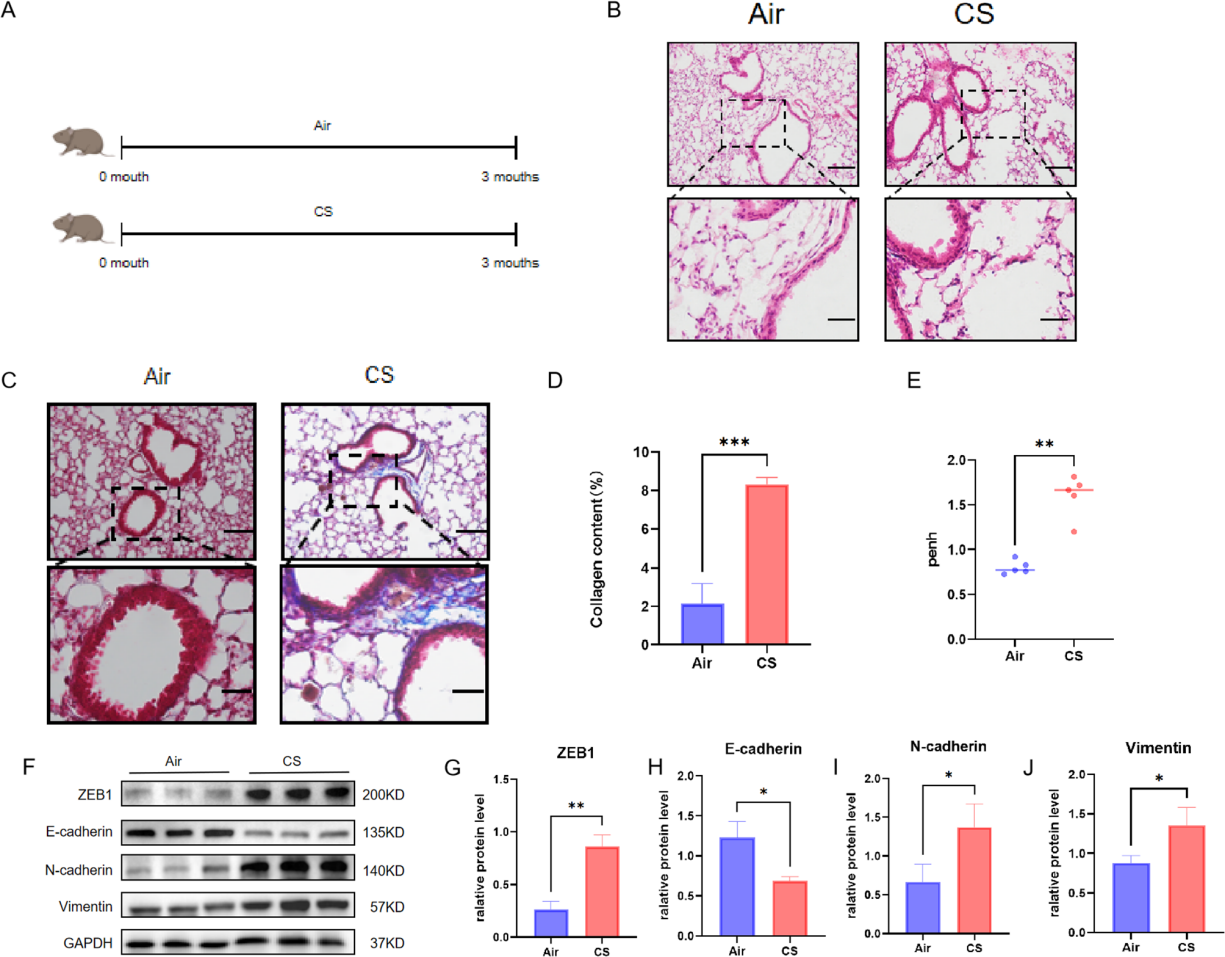
Airway remodelling was triggered in CS-induced experimental COPD. (**A**) Schematic diagram of male C57BL6J mice exposed to air or CS treatment. (**B**) Conducting H&E staining on lung sections, scale bar, 100 μm (up). (**C**) Conducting Masson staining on lung sections assessed lung morphology. Nuclei: black; collagen: blue; cytoplasm/epithelial: red. Scale bars, 100 μm(up). (**D**) Quantitative staining of Masson content in control group (*n* = 3) mice and CS exposed group mice(*n* = 3). (**E**) The Penh content in CS-exposed mice (*n* = 5) increased compared to the control group (*n* = 5). (**F**-**J**) Western blot showed the ZEB1, E-cadherin, N-cadherin, and Vimentin in the lung tissue of CS-exposed mice and the control group (*n* = 3). The data are the mean ± SD. * *P* < 0.05, ** *P* < 0.01,*** *P* < 0.001

Next, we established an experimental COPD mouse model (Fig. 2A). After 3 months of exposure to CS, BALB/c mice developed COPD, exhibiting a phenotype of airway remodelling, collagen deposition and airway thickening (Fig. 2B, C and D). Compared with the control group, the Penh content in CS-exposed mice increased (Fig. 2E). In addition, the western blot showed a decrease in E-cadherin and an increase in ZEB1, N-cadherin, and Vimentin in the lung tissue of CS-exposed mice compared with the control (Fig. 2F and J).

### GLUT3 increased in mice exposed to CS and BEAS-2B cells treated with CSE

**Fig. 3:**
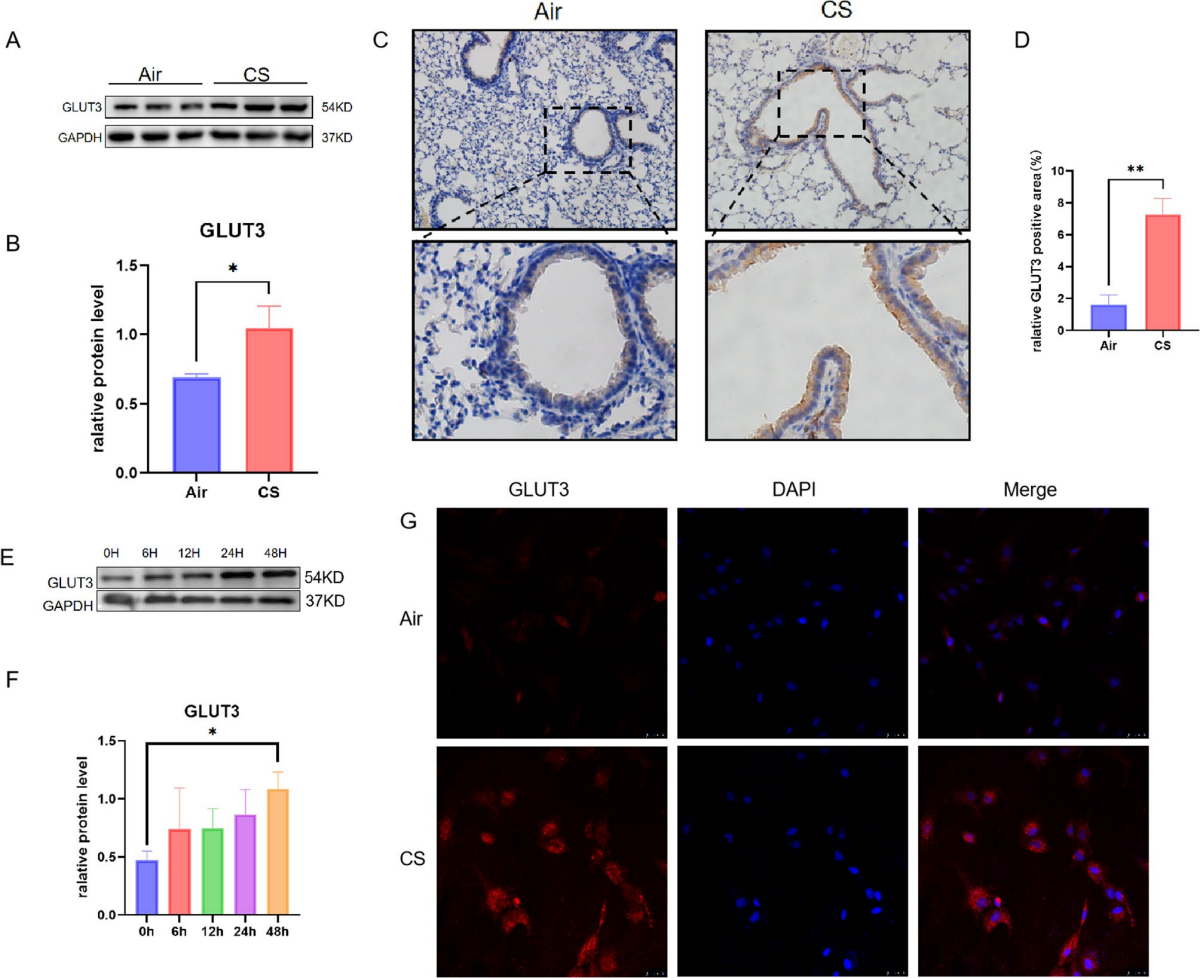
GLUT3 increased in mice exposed to CS and BEAS-2B cells treated with CSE. (**A**) and (**B**) Western blot showed the expression levels of GLUT3 and GAPDH in the lungs of mice exposed to air (*n* = 3) and CS (*n* = 3). (**C**) and (**D**) Representative GLUT3 immunostaining (brown staining) was performed in the airways of mice exposed to air (*n* = 3) and CS (*n* = 3), Scale bars, 100 μm(up). (**E**) and (**F**) Western blot detected the time course of GLUT3 expression in the 5% CSE response. (**G**) Immunofluorescence results showed that after 48 h of treatment with 5% CSE in BEAS-2B cells, GLUT3 protein expression was enhanced, scale bar, 100 μm. The data are the mean ± SD. * *P* < 0.05, ** *P* < 0.01,*** *P* < 0.001

We evaluated the expression levels of GLUT3 in the lung tissues of control and experimental COPD mice to investigate the potential involvement of GLUT3 in the pathogenesis of COPD. Western blot analysis showed that the GLUT3 in the experimental COPD mice was higher than in the lungs of mice exposed to air (Fig. 3A and B). In addition, immunohistochemical staining showed an increase in GLUT3 in the bronchial epithelial cells of mice (Fig. 3C and D). Western blot showed a time-dependent increased expression of GLUT3 after 48 h CSE exposure (Fig. 3E and F). Conducting immunofluorescence staining in BEAS-2B cells showed enhanced fluorescence staining of GLUT3 after 48 h of CS exposure (Fig-3G).

### GLUT3 played a crucial role in airway remodelling in experimental COPD

**Fig. 4:**
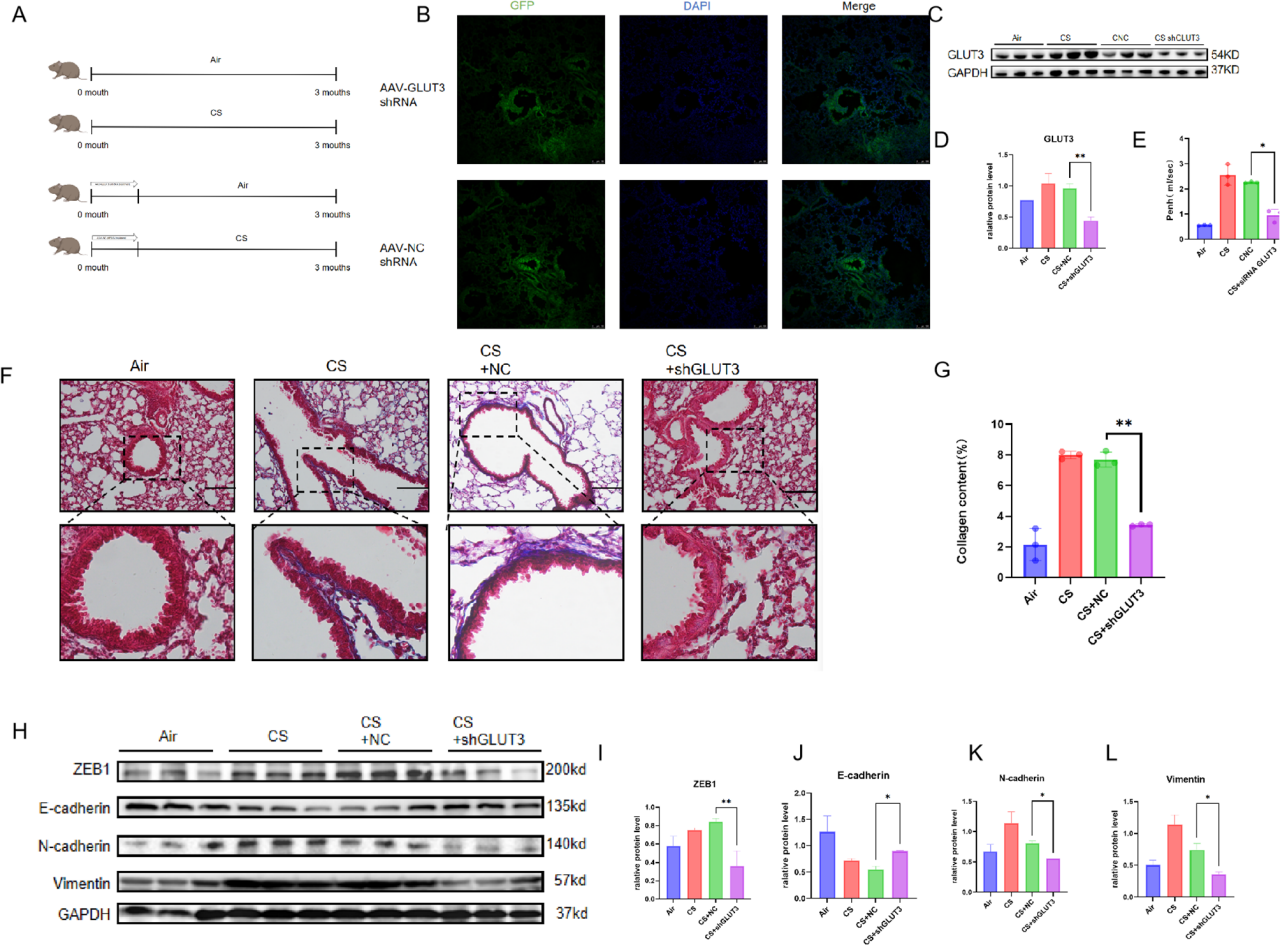
GLUT3 played a crucial role in airway remodelling in experimental COPD (**A**) The Schematic chart of Male C57BL6J mice treatment (**B**) The efficiency of AAV infection in mouse lung tissues is shown in immunofluorescence images, scale bars, 50 μm. (**C-D**) Western blot showed the expression of GLUT3 and GAPDH in the lungs of mice exposed to air and CS (*n* = 3 per group). (**E**) Pulmonary function was represented as Penh (*n* = 3 per group) (**F**) Performing Masson staining on lung tissue assessed lung morphology. Nuclei: black; collagen: blue; cytoplasm/epithelial: red. Scale bars, 200 μm. (**G**) Quantitative staining of Masson content in mice (*n* = 3 per group). (**H-L**) Western blot showed ZEB1, E-cadherin, N-cadherin, and Vimentin in the lungs of mice (*n* = 3 per group). The data are the mean ± SD. * *P* < 0.05, ** *P* < 0.01,*** *P* < 0.001

Next, we investigated the functional role of GLUT3 in the pathogenesis of experimental COPD. We used the AAV9-GLUT3 inhibitor to reduce GLUT3 expression in the mice model before being exposed to CS (Fig. 4A-C). Penh decreased in AAV9 shRNA-GLUT3-inhibitor-treated mice in contrast to the NC shRNA-treated mice (Fig. 4D). In vitro experiments showed that Masson staining decreased collagen deposition in AAV9 shRNA-GLUT3-inhibitor-treated mice in contrast with the NC shRNA-treated mice (Fig. 4E and F). Here, GLUT3 reduction significantly increased E-cadherin in the lung tissue of experimental COPD mice and reduced the expression of ZEB1, N-cadherin, and Vimentin (Fig. 4G-K). These data support the important contribution of reducing GLUT3 expression in improving airway remodelling in experimental COPD.

### GLUT3 enhanced CSE-induced EMT in BEAS-2B cells

**Fig. 5:**
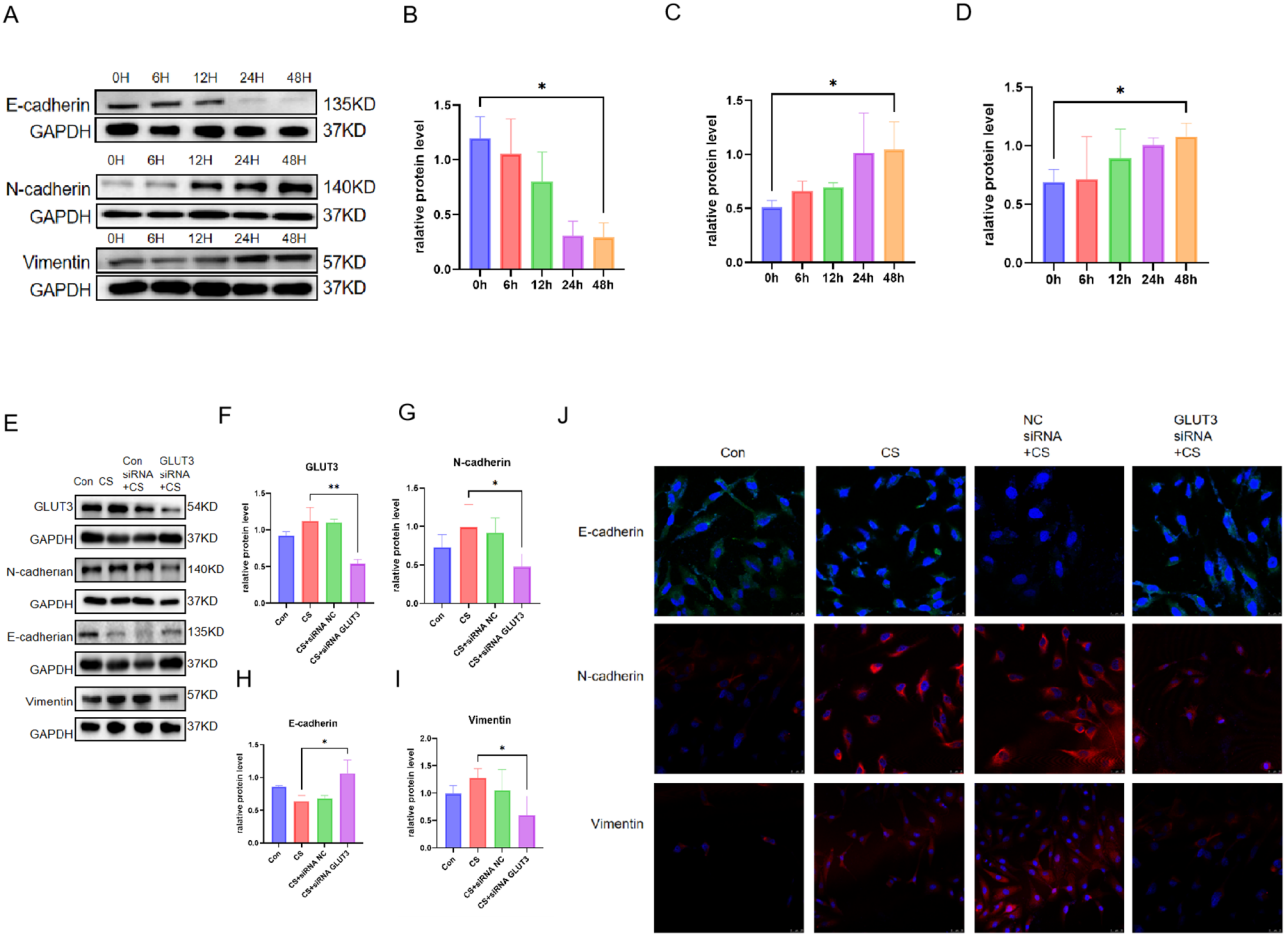
GLUT3 enhanced CSE-induced EMT in BEAS-2B cells. (**A**-**D**) Western blot showed the expression of E-cadherin, N-cadherin, and Vimentin after 5% CSE treatment of BEAS-2B cells for 48 h, (**E**-**I**) Transfecting BEAS-2B cells with NC siRNA or GLUT3 siRNA before control or 5% CSE treatment. The expression of GLUT3, N-cadherin, E-cadherin, and vimentin protein in BEAS-2B cells before and after transfection was detected by western blots. (**J**) Immunofluorescence detection showed the expression of E-cadherin, N-cadherin, and vimentin proteins in BEAS-2B cells. The data are the mean ± SD. * *P* < 0.05, ** *P* < 0.01

We further confirmed the involvement of GLUT3 in airway remodelling in COPD induced by smoking. Western blot showed that after 48 h of BEAS-2B cells exposed to CSE, the expression of N-cadherin, Vimentin, and E-cadherin protein increased in a time-dependent manner (Fig. 5A and D). We successfully transfected GLUT3 siRNA into BEAS-2B cells to investigate whether GLUT3 can regulate airway remodelling. Subsequently, Western blot showed that E-cadherin was increased, and N-cadherin and vimentin protein were decreased in BEAS-2B cells transfected with GLUT3 siRNA (Fig. 5F and I). The increase in E-cadherin protein and the decrease in N-cadherin and Vimentin indicate an improvement in airway remodelling. Cell immunofluorescence showed that in BEAS-2B cells transfected with GLUT3 siRNA, the expression of E-cadherin protein was enhanced compared with those in BEAS-2B cells treated with CSE. In contrast, N-cadherin and vimentin protein expression decreased (Fig. 5J).

### GLUT3 reduction down-regulated the NF-kB/ZEB1 pathway in CSE-treated BEAS-2B cells

**Fig. 6:**
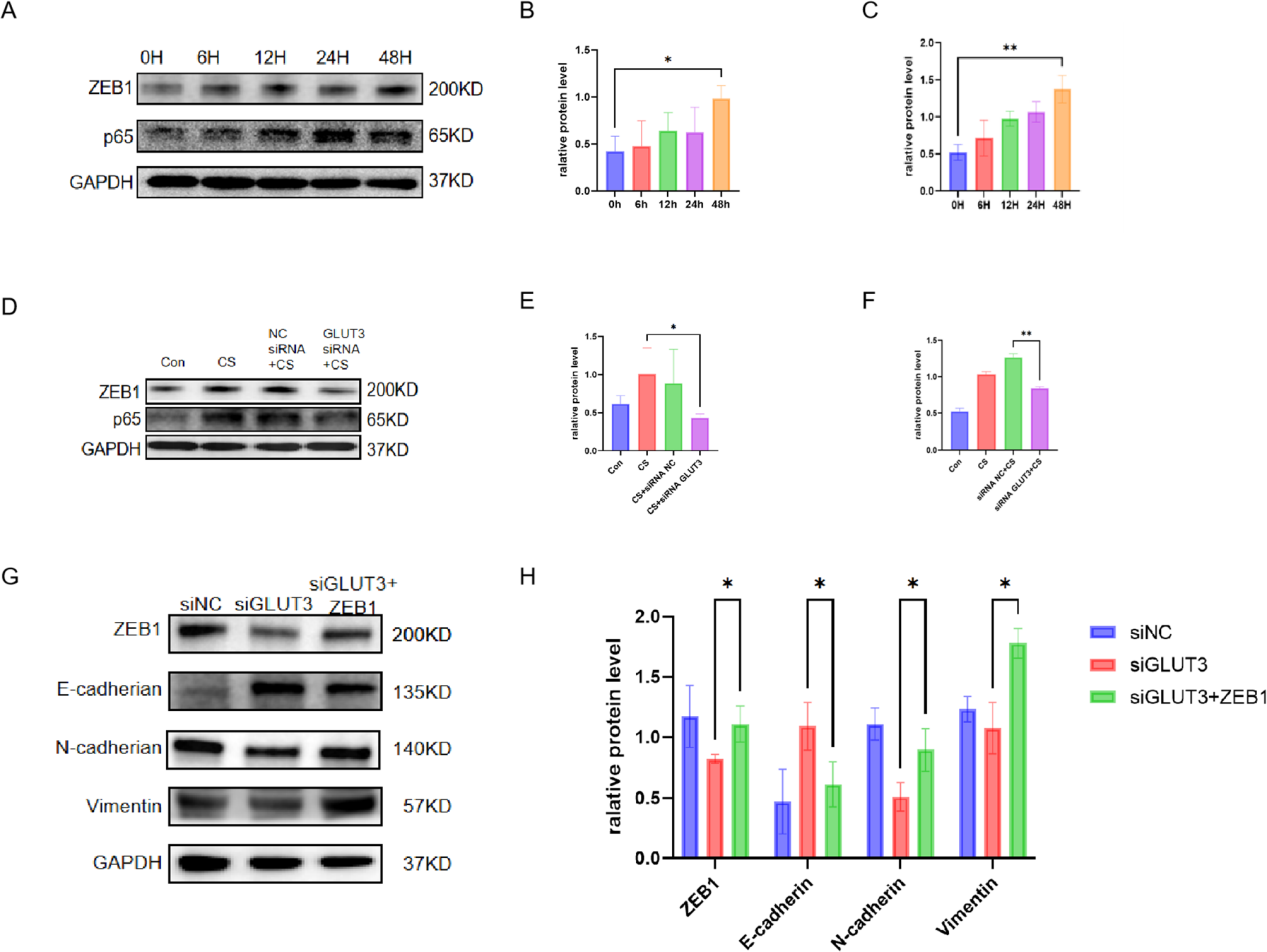
GLUT3 reduction down-regulated the NF-kB/ZEB1 pathway in CSE-treated BEAS-2B cells (**A**-**C**) The time-dependent expression of p65 and ZEB1 after treating BEAS-2B cells with 5% CSE for 48 h by western blot. (**D**-**F**) Before 5% CSE treatment, BEAS-2B cells were transfected with NC or GLUT3 siRNA. Western blot detected p65 and ZEB1 in BEAS-2B cells before and after transfection. (**G-H**) Western blot detected the expression of ZEB-1, E-cadherin, N-cadherin, and Vimentin. The data is average ± standard deviation (*n* = 3)* *P* < 0.05, ** *P* < 0.01

NF-κB is essential in the gene regulatory program that changes cell morphology, a process known as EMT [21–23]. The NF-κB/ZEB1 pathway has been proven to reduce E-cadherin expression in cells [24]. Therefore, to investigate the role of GLUT3 in the NF-κB/ZEB1 pathway, we analysed the expression of p65 and ZEB1 in GLUT3-reduced cells. After BEAS-2B cells were exposed to CSE for 48 h, we observed that p65 and ZEB1 protein expression increased in a time-dependent manner (Fig. 6A and C). GLUT3 reduction also reduced the expression of p65 and ZEB1 (Fig. 6D and F).

Furthermore, we overexpressed zeb1 through plasmid transfection and then reduced the expression of GLUT3. This process resulted in a significant decrease in E-cadherin and an increase in N-cadherin and Vimentin (Fig. 6G-H). These data indicated that the expression of ZEB1 is required for EMT in BEAS-2B cells regulated by GLUT3. In summary, our research findings demonstrate that GLUT3 may regulate the EMT signalling pathway by the NF-κB/ZEB1 pathway.

## Discussion

COPD, as a chronic respiratory disease caused by gene-environment interactions, has become a global public health challenge due to its high incidence and mortality rate [25]. Epithelial-mesenchymal transition (EMT) caused airway remodelling, profoundly affecting COPD pathology. However, there is currently no clinically effective treatment method to prevent pathological changes in the airway of COPD. In our research, we explored the potential role of GLUT3 in the airway in COPD and its possible molecular mechanisms. Through our analysis of two GEO datasets, GLUT3 expression in COPD was higher than that in the control, and GLUT3 was up regulated in the lung tissues of patients with COPD compared to the control group.

In addition, GLUT3 is up-regulated in the bronchial epithelial cells of CS-exposed mice and BEAS-2B cells exposed to CSE. Our findings suggest that GLUT3 plays a part in the occurrence and development of COPD. Further studies indicated that the reduction of GLUT3 reduced CSE-induced EMT in BEAS-2B cells and relieved airway remodelling in experimental COPD. Regarding mechanism, CSE exposure may lead to NF-κB/ZEB1 signalling activation, inhibited by GLUT3 reduction.

Previous studies have found that the occurrence of EMT induced by CS in airway epithelial cells was an important reason for the increase in small airway fibroblasts, excessive deposition of ECM, and subsequent airway remodelling [25, 26]. In this study, we investigated the effects of cigarette smoke on the occurrence of EMT in vitro and in vivo. The results are consistent with other studies, indicating that cigarette smoke led to the occurrence of EMT in CS-exposed mice. At the same time, our in vitro experiments also confirmed that CSE treatment can induce EMT in BEAS-2B cells. Therefore, EMT, which plays a key role in epithelial cell development during airway remodelling, has always been considered an important target for the treatment of COPD.

Glucose transporters are the first step in mediating glucose metabolism, and GLUT3 is crucial for glucose uptake in airway epithelial cells [13]. Recent studies have shown that GLUT3 expression is significantly increased during EMT, which plays a significant role in the poor prognosis of various cancers [18]. It also plays an important intrinsic role in maintaining the immune defence characteristics of the airway while maintaining the glucose homeostasis of the entire airway epithelial cells [19]. Previous studies have shown that GLUT3 is considered a transcriptional target of ZEB1, playing a significant role in lung cancer when tumour cells lose their epithelial features and become more aggressive, promoting lung cancer metastasis [14]. At the same time, some studies have found that GLUT3 may significantly affect the progression of head and neck squamous cell carcinoma and colorectal cancer cells by regulating EMT [18, 27]. Our previous experiment found that GLUT3 was enhanced in the alveolar macrophages of patients with COPD. The expression of GLUT3 was also increased in THP-1 macrophages (THP-M cells) exposed to CSE [28]. However, the effect of GLUT3 in airway epithelial cells of patients with COPD has not been studied to date. In our study, we first observed that the expression of GLUT3 may be involved in COPD. Due to the important relationship between GLUT3 and metabolic reprogramming, further in-depth research is required to verify the relationship between GLUT3 and glucose metabolic reprogramming in bronchial epithelial cells.

Although the mechanism of EMT in COPD has not been fully elucidated, the NF-κB/ZEB1 signalling was believed to be involved in EMT in COPD [29]. Several transcription factors have been confirmed as key regulators of EMT, such as Snail, Twist, Slug, and ZEB-1 [30]. In Jian Sun’s study, the expression of ZEB1 was increased in COPD. NF-κB/ZEB1 not only plays a core role in inflammation but also plays an important role in controlling the gene regulatory program that drives changes in cell state, a process known as EMT [31]. Previous high throughput RNA-Seq studies of normal human airway cells found that the core regulatory network of EMT in normal epithelial cells overlapped with the NF-κB pathway [22]. Using selective knockouts, NF-κB signalling drove the transition of epithelial cells to mesenchymal phenotype in bronchiolar-derived basal cell progenitors [32]. These findings showed that NF-κB signalling in the airway produced EMT in vivo. We identified NF-κB/ZEB-1 as an effective pathway of these induced phenomena to suggest the mechanism of how GLUT3 modulated EMT in BEAS-2B cells. It is well known that NF-κB/ZEB-1 is involved in cancer invasion in various tumours, including the process of EMT in breast cancer [24]. Recently, it has been reported that over-expression of GLUT3 up-regulated EMT-related genes such as N-cadherin, Vimentin, and ZEB1 down-regulated the expression of E-cadherin in colorectal cancer [19]. This study found that with GLUT3 knock-down, the NF-κB/ZEB-1 pathway was down-regulated, and E-cadherin expression was up-regulated in EMT, consistent with previous studies. However, some studies have also reported that the expression of GLUT3 was induced during the EMT of non-small cell lung cancer (NSCLC) [14]. The transcription factor ZEB1 can directly bind to GLUT3 to regulate the mRNA of GLUT3, achieving the regulation of GLUT3 expression. Therefore, a complex network relationship between GLUT3 and ZEB1 may need further research and discovery. In addition, Dong-Min Yu found that GLUT3 interacts directly with RAS and regulates PAK activation and IL-4R endocytosis. GLUT3 acted on the nucleus through endocytosis [33]. This process is significant for the M2 polarisation of macrophages by regulating IL-4/STAT6 signalling. We believe that GLUT3 may activate the NF-κB/ZEB1 pathway through endocytosis. Therefore, further studies are required to elucidate the relationship between GLUT3 and NF-κB.

Our study also has some limitations. Firstly, BEAS-2B is a commercial cell line rather than a primary cell line, which may affect the conclusion of this article. Previous data suggests that there are differences in the response of bronchial epithelial cells from COPD patients and those from healthy patients[34], which may affect the role of GLUT3. It is likely that airway fibrosis and emphysema underlie the irreversible nature of airway obstruction. Our findings are related to peribronchial fibrosis. Meanwhile, in our research, HE staining did not show significant improvement in emphysema of AAV9 shRNA-GLUT3-inhibitor-treated mice in contrast with the NC shRNA-treated mice. In addition, the GOLD stages of the COPD patients are mainly GOLD 3 and 4. We did not describe or discuss the GOLD stages of COPD, which is a disadvantage.

**Fig. 7:**
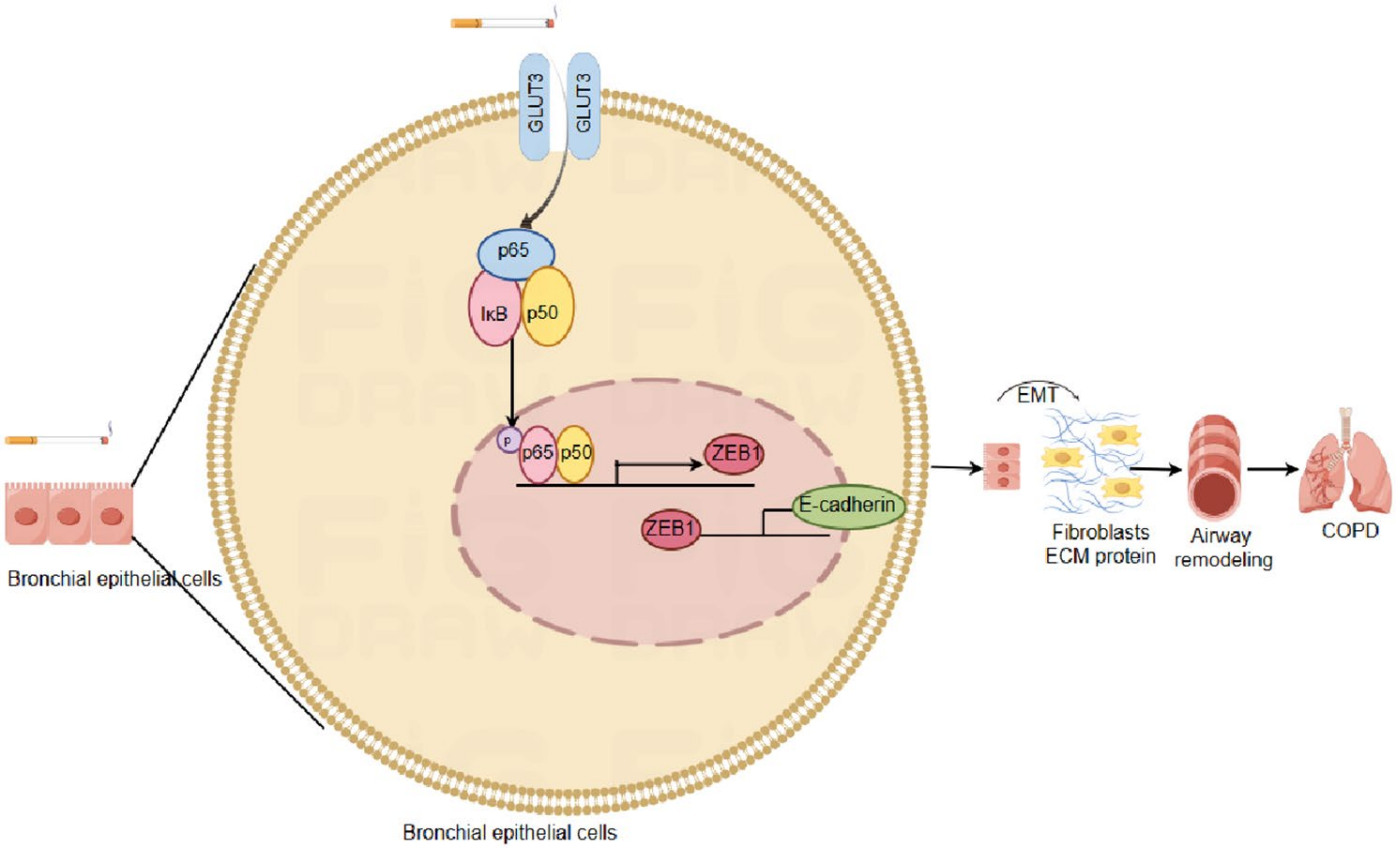
Schematic representation of plausible mechanism shows the potential role of GLUT3 in COPD. GLUT3 is positively regulated in bronchial epithelial cells and GLUT3-mediated cigarette smoke induced epithelial-mesenchymal transition by up-regulating the NF-κB/ZEB1 pathway, inducing airway epithelial cells to lose their epithelial characteristics with the loss of polarity and junctional proteins. Airway epithelial cells acquire mesenchymal features such as a spindle shape and to the ability to migrate and secrete matrix proteins. Finally, the extracellular matrix deposition causes airway remodelling, ultimately leading to COPD

In summary, our study demonstrated that the up regulation of GLUT3 was closely related to the occurrence of airway remodelling in COPD. GLUT3 played an important role in promoting EMT in bronchial epithelial cells and caused excessive deposition of ECM, which may be related to the activation of the NF- κB/EMT pathway. In summary, these findings indicated the potential research value of GLUT3 in diagnosing and treating COPD (See Fig 7) give link.

## Data Availability

No datasets were generated or analysed during the current study.
